# Off‐Label use of a lumen‐apposing metal stent for refractory pyloric stricture in a toddler

**DOI:** 10.1002/jpr3.70286

**Published:** 2026-11-02

**Authors:** Hamza Hassan Khan, Prateek Wali, Sumant Inamdar

**Affiliations:** ^1^ Section of Gastroenterology, Hepatology and Nutrition, Department of Pediatrics University of Arkansas for Medical Sciences and Arkansas Children's Hospital Little Rock Arkansas USA; ^2^ Section of Gastroenterology, Hepatology and Nutrition, Department of Internal Medicine University of Arkansas for Medical Sciences Little Rock Arkansas USA

**Keywords:** endoscopic stenting, gastric outlet obstruction, pediatric endoscopy

A 21‐month‐old girl with alpha‐thalassemia trait presented with hematemesis and melena following an upper respiratory infection. Her symptoms improved with high‐dose twice‐daily proton pump inhibitor therapy, and she was discharged. Ten days later, she re‐presented with recurrent vomiting and hematemesis. An upper gastrointestinal series demonstrated impaired passage of contrast across the pylorus and narrowing of the pyloric channel (Figure 1A). Upper endoscopy showed marked pyloric inflammation with a fixed stricture (Figure 1B). Mucosal biopsies were nonspecific and negative for Helicobacter pylori, and there was no history of nonsteroidal anti‐inflammatory drug use. Endoscopic balloon dilation was performed (starting at 6 mm and up to 10 mm), followed by repeat dilations 3 weeks (up to 12 mm) and 8 weeks (starting at 8 mm and up to 12 mm) later. Because the stricture persisted despite serial dilation, a 15 × 15‐mm lumen‐apposing metal stent (AXIOS; Boston Scientific) was placed across the pylorus during the third session (Figure 1C,D) and removed 3 months later (Figure 1E). No stent‐related adverse events occurred. At 4‐month follow‐up after stent removal, she remained asymptomatic and tolerated a regular solid diet. Although the exact etiology remained uncertain, a postinfectious inflammatory pyloric stricture was considered, with the lumen‐apposing metal stent serving as a bridge to maintain pyloric patency while the underlying inflammation resolved. This case demonstrates the successful off‐label use of a lumen‐apposing metal stent for a refractory inflammatory pyloric stricture in a toddler and suggests a potential role for temporary stenting in maintaining luminal patency during resolution of severe pyloric inflammation.

**Figure 1:**
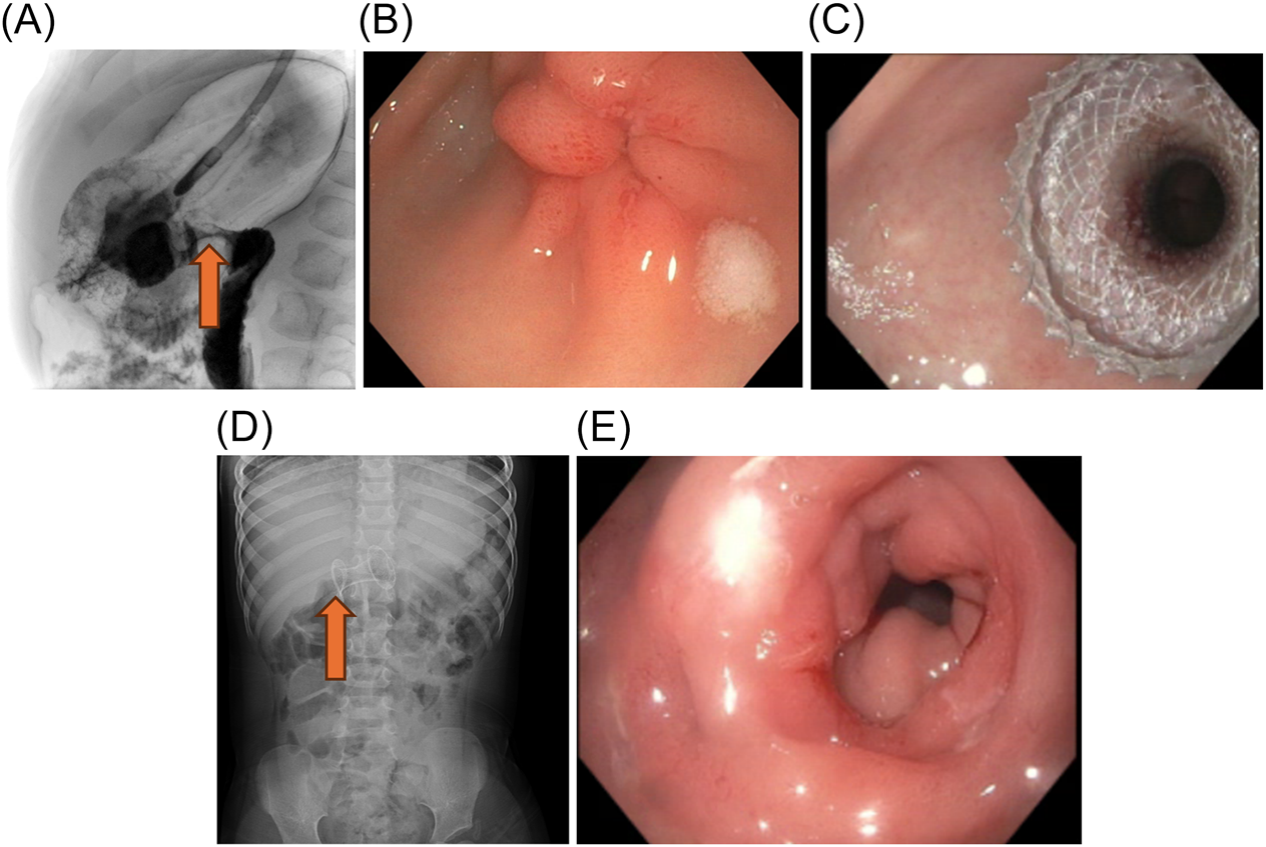
(A) Upper gastrointestinal contrast study demonstrating impaired passage of contrast across the pylorus with marked narrowing of the pyloric channel (arrow). (B) Endoscopic view showing severe inflammatory narrowing of the pylorus with a fixed stricture before dilation. (C). Endoscopic view after deployment of a 15 × 15‐mm lumen‐apposing metal stent across the pyloric stricture, with the proximal flange positioned within the gastric antrum. (D) Supine anteroposterior abdominal radiograph demonstrating the pyloric stent (arrow) in appropriate position with a nonobstructive bowel gas pattern. (E) Endoscopic view following stent removal demonstrating a widely patent pylorus.

## CONFLICT OF INTEREST STATEMENT

The authors declare no conflicts of interest.

## ETHICS STATEMENT

Informed consent was obtained from the mother (legal guardian) of the patient.

